# Chats about Medical Museums

**Published:** 1887-03-05

**Authors:** 


### Chats about Medical Museums.

By One of the Crowd.

The medical museums of London are among its most deeply interesting exhibitions. They are not ordinarily accessible to the general public, and perhaps it is just as well that they are not. The knowledge to be derived from them by persons who are not educated in scientific matters would be, in many cases at least, but a dubious kind of gain, and to not a few persons would, perhaps, prove to be of more harm than good in more ways than one. Nevertheless they are all of them intensely interesting, and all of them, more or less, rich in objects of what may be called popular curiosity and instruction.

The largest of all the medical museums of London—indeed, one of the finest in the world—is the great central collection at the Royal College of Surgeons, Lincoln's Inn Fields. One may go in here and see himself taken to pieces, may examine every little bone and muscle, and vein and artery of his body. He may trace his physical history from the very earliest stages of his existence up to maturity, and may see almost all the ills to which his flesh is heir, illustrated by models in wax, or by specimens preserved in spirits. He may turn to the catalogues, and trace the symptoms of progressing disease, of maladies and malformations slowly creeping on, and he will be a fortunate individual if he can leave the building without bearing away with him a dismal and depressing suspicion that he himself can detect some of the symptoms he has been reading about, and is in a fair way to become a subject for another model, or to have some day one of his limbs bottled up in spirits for the perpetual instruction of "the Faculty."

There are in fact three museums at the College of Surgeons—the western, the middle, and the eastern museum, each room having two galleries, one above the other, all round it, the whole being crowded with objects illustrating human and comparative anatomy in health and disease. Nearly all round the ground-floor of the great hall constituting the western museum, are glass-cases thronged with skulls and skeletons of people from all corners of the earth—Polynesians and Turks, Russians and Indo-Malayans, Austrians and Egyptians, Patagonians and Backwood Indians, they are all here, and stripped of their outward trappings, their paint and feathers and other finery, and the flesh with all its multifarious demands, and varying forms and phases, the whole mixed mob look uncommonly like brothers in adversity. They have come from all the ends of the earth, and here they stand grinning at each other as though rather tickled at the discovery that after all they are so much alike, that, apart from mere surface differences, they have so much in common with each other. Among the heads here is one of an Egyptian female of ancient times. It was found in the neighbourhood of Thebes in a state of excellent preservation, and still looks as pert if not as pretty as it may have done when she lent the Israelites her trinkets of gold or silver to get rid of them out of the land.

There are a good many examples of the various artificial modes adopted by different nations for the preservation of dead bodies. This Egyptian head is one, and there is another, the body of a male Peruvian found in a sepulchre in Peru, at a spot at which native tradition tells of a voluntary sacrifice of life by a noble belonging to an order immediately below that of the Incas or members of the royal family. In fact, he was a duke, and when he buried himself alive, or otherwise effected the happy despatch, probably little thought that there was yet before him in the body a distinguished career in a land far away over the ocean. The catalogue seems to assume the identity of the mummy with the self-sacrificing Curaca, and tells us that it was found ten feet below the surface in a dry calcareous soil, to which and to the dryness of the air is to be attributed its preservation in an undecomposed state.

Among hundreds of other skeletons is that of the notorious Jonathan Wild, looking as grimly jocund as though he had never sworn any poor wretch's life away, and never had been hanged at Tyburn and pelted by an exasperated crowd to the last moment of his existence. The largest skeleton of the collection is that of Charles O'Brian, the famous Irish giant. When he died, about a hundred years ago, he measured eight feet four inches, and here as a skeleton he stands seven feet seven inches. Poor O'Brian in having his bones thus exhibited in a glass-case is, one cannot help thinking, rather a hardly-used skeleton. He seems to have been so sick of publicity in his lifetime that just before he died he is said to have begged that his remains might be taken out to sea, and dropped down into the depths and forgotten. Rather too bad after that to strip his bones bare, to set him up here on show with a tablet appended, setting forth not only his name and nationality, his age and height, but also putting on perpetual record his most discreditable little weakness. He is said to have died at the age of twenty-two from excessive drinking. Just behind him is the bony framework of a dwarf, Caroline Crachami, exhibited in London in 1824, and measuring now just twenty inches high. This appears to be the smallest adult skeleton in the collection, and it, of course, looks all the smaller from its proximity to O'Brian, with his seven feet seven inches, and another nameless individual beside him standing six feet nine inches, and who in the flesh must have been over seven feet. The smallest human skeleton in this collection among those actually born into this troublesome world is that of a child about sixteen inches and a half in length. This is the framework of a new-born infant, but there is a whole series of skeletons of earlier stages of development, running by small gradations down to a tiny object perhaps an inch in length. This curious series is contained in a glass-case in the central portion of the floor, over which is extended the enormous skeleton of a full-grown whale, captured off the coast of South Greenland, and brought from Copenhagen to England in 1864, the first of its species ever seen in this country. Underneath this whale there are glass-cases, in which not only is every bone of a human frame exhibited individually, but the bones themselves are, so to speak, taken to pieces, and their structure displayed.

Some of the other objects of popular interest in this wonderful collection must be referred to in another article.

